# Supplementary material for: Predicting visual function by interpreting a neuronal wiring diagram
Source: Nature. 2024 Oct 2;634(8032):113–23. doi: 10.1038/s41586-024-07953-5 (PMC11446822; doi:10.1038/s41586-024-07953-5)
Supplement: Supplementary file 6 — CRF and ERF predictions for individual TmY4 and TmY9 cells. Analogous to Supplementary Data 3, but for TmY target types. Shown are the top four monosynaptic pathways, the strongest pathway passing through each of the top ten intermediary types (ranking from Extended Data Fig. 7), and the trisynaptic pathway Tm1–TmY–Dm3–TmY (see the section entitled Prediction of spatial normalization). [file 41586_2024_7953_MOESM6_ESM.zip › DataS4/TmY4/720575940616716957.pdf]

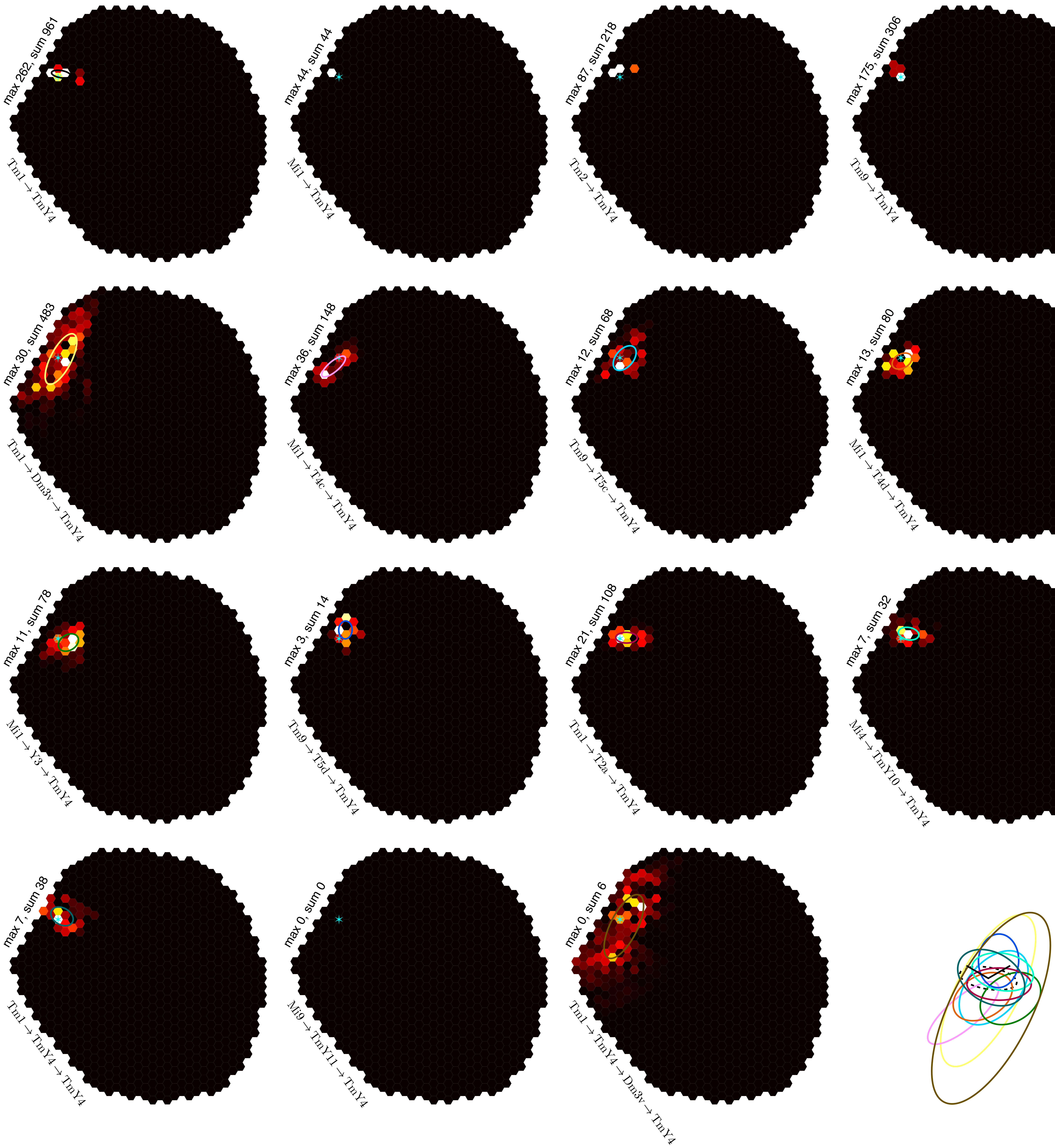
max 262, sum 961
Tm1 → TmY4
max 44, sum 44
Mi1 → TmY4
max 87, sum 218
Tm2 → TmY4
max 175, sum 306
Tm9 → TmY4
max 30, sum 483
Tm1 → Dm3v → TmY4
max 36, sum 148
Mi1 → T4c → TmY4
max 12, sum 68
Tm9 → T5c → TmY4
max 13, sum 80
Mi1 → T4d → TmY4
max 11, sum 78
Mi1 → Y3 → TmY4
max 3, sum 14
Tm9 → T5d → TmY4
max 21, sum 108
Tm1 → T2a → TmY4
max 7, sum 32
Mi4 → TmY10 → TmY4
max 7, sum 38
Tm1 → TmY4 → TmY4
max 0, sum 0
Mi9 → TmY11 → TmY4
max 0, sum 6
Tm1 → TmY4 → Dm3v → TmY4
